# Exploring the Pathophysiology of Long COVID: The Central Role of Low-Grade Inflammation and Multisystem Involvement

**DOI:** 10.3390/ijms25126389

**Published:** 2024-06-09

**Authors:** Evgenii Gusev, Alexey Sarapultsev

**Affiliations:** Institute of Immunology and Physiology, Ural Branch of the Russian Academy of Science, 620049 Ekaterinburg, Russia; gusev36@mail.ru

**Keywords:** chronic inflammation, diagnostic strategies, long COVID, low-grade inflammation, inflamm-aging, PASC, pathophysiology, personalized medicine, post COVID-19 condition, systemic inflammation

## Abstract

Long COVID (LC), also referred to as Post COVID-19 Condition, Post-Acute Sequelae of SARS-CoV-2 Infection (PASC), and other terms, represents a complex multisystem disease persisting after the acute phase of COVID-19. Characterized by a myriad of symptoms across different organ systems, LC presents significant diagnostic and management challenges. Central to the disorder is the role of low-grade inflammation, a non-classical inflammatory response that contributes to the chronicity and diversity of symptoms observed. This review explores the pathophysiological underpinnings of LC, emphasizing the importance of low-grade inflammation as a core component. By delineating the pathogenetic relationships and clinical manifestations of LC, this article highlights the necessity for an integrated approach that employs both personalized medicine and standardized protocols aimed at mitigating long-term consequences. The insights gained not only enhance our understanding of LC but also inform the development of therapeutic strategies that could be applicable to other chronic conditions with similar pathophysiological features.

## 1. Introduction

On 5 May 2023, the head of the World Health Organization (WHO) declared the global health emergency caused by COVID-19 over. However, the disease continues to pose a global threat due to its long-term consequences [1]. Known as long COVID (LC) or Post COVID-19 condition [2], which is classified under codes U09.9 (ICD-10) and RA02 (ICD-11), it is also referred to by various other terms including Chronic COVID Syndrome [3], Late Sequelae of COVID-19 [4], Long-haul COVID [5], Post-acute Sequelae of SARS-CoV-2 Infection (PASC) [6], and Post-COVID Syndrome [7]. The global prevalence of LC is not precisely known, and attempts to estimate it have yielded heterogeneous results [8]. Recent global meta-analyses suggest that the cumulative prevalence of LC ranges from 9% to 63% in COVID-19 survivors, approximately six times higher than that of similar post-viral conditions [9].

LC represents a multisystem pathology lacking clear clinical boundaries and standardized patient management protocols. This complicates the description of its pathogenesis. Initially, it is essential to approach LC from a methodological perspective of general pathology, which considers three levels of human pathology assessment:

- Personalized medicine [10] takes into account individual disease characteristics, as well as the genotype and phenotype of the patient, and environmental factors.
- Generic clinical definition protocols such as the ICD-11 taxonomy, which includes approximately 85,000 categories, and the MalaCards integrated database, which lists about 20,000 disease names [11].
- Evaluations of pathologies based on models of general pathological processes, including various types of canonical and non-classical inflammation. These models classify the pathogenesis of diseases into broader disease categories.
- Conceptual syndromes serve as surrogate forms of general pathological processes and clinical definitions. They temporarily bridge gaps in general pathology knowledge to support clinical practice needs. For example, the syndrome of systemic inflammatory response preceded the adoption of Sepsis-3 [12] and the current clinical interpretations of “cytokine storm” and systemic inflammation. “Post-COVID syndrome” may also be categorized under such marginal definitions.

The primary aim of this article is to substantiate the critical role of local and systemic low-grade inflammation—a non-classical inflammatory process—as a fundamental pathological mechanism in LC. The following objectives will guide this exploration:

- Provide a general characterization of LC (Section 2).
- Summarize methodological approaches and limitations (Section 3).
- Discuss the main theories of typical pathological processes and the role of cellular and tissue stress as a foundation for pathogenetic models of various human diseases (Section 4).
- Describe low-grade inflammation from the perspective of general pathology and its pathogenetic role in viral infections (Section 5).
- Define the role of low-grade inflammation in various clinical manifestations of LC (Section 6).
- Summarize the study’s findings (Section 7).

## 2. General Characterization of Long COVID

By the end of 2022, over 200 symptoms and at least 50 clinical conditions persisting for more than three months, which directly or indirectly define LC, have been identified [13]. Notably, a meta-analysis has demonstrated a significantly increased risk of long-term fatigue (RR 1.72, CI 1.41–2.10), dyspnea (RR 2.60, CI 1.96–3.44), memory difficulties (RR 2.53, CI 1.30–4.93), and concentration challenges (RR 2.14, CI 1.25–3.67) associated with SARS-CoV-2 infection [14]. LC encompasses all body systems and presents diverse and often unstable symptom complexes, complicating its clinical definition. It is premature to categorize LC as chronic COVID-19, as persistent SARS-CoV-2 has not been confirmed in all cases [15].

LC’s development does not necessarily correlate with the severity of acute COVID-19, nor with ongoing SARS-CoV-2 presence post-hospital discharge. Alarmingly, LC also affects children, including those who were asymptomatic, leading to persistent symptoms like dyspnea, fatigue, and cognitive impairments lasting at least six months [15]. Reinfections with SARS-CoV-2 may increase LC likelihood, though risks are lower if subsequent infections are mild or asymptomatic [16,17]. Although LC can affect any part of the body, children with this condition more frequently experienced symptoms related to the head and neck, particularly loss of smell and taste. Brain fog affected between 2% to 44% of children with LC. Heart problems were rare, but some children continued to have symptoms, including increased heart rate. There was also a higher incidence of mental health disorders, including anxiety [17,18].

Perumal et al. have highlighted several key pathogenetic mechanisms and associated risk factors for LC, such as persistent virus and SARS-CoV-2 antigen presence, microclot formation in the bloodstream, widespread autoimmunity, reactivation of latent viruses, mast cell activation, chronic low-grade systemic inflammation, significant disruptions in the gastrointestinal tract leading to dysbiosis and intestinal barrier dysfunction, extensive changes in the central nervous system (CNS), metabolic dysfunction or bioenergetic failure, and autonomic dysfunction [19]. Identified risk factors include gender, the presence of more than five early symptoms including dyspnea, prior psychiatric disorders, and specific biomarkers such as D-dimer, CRP, and lymphocyte count [15]. However, the data varies, with other authors identifying female gender, active smoking, and comorbidities as risk factors for the LC development, while age does not appear to be a significant factor [20,21,22].

Genetic polymorphisms in LZTFL1 rs10490770, LZTFL1 rs11385942, LZTFL1 rs17713054, NADSYN1 rs12785878, PLXNA4 rs1424597, IL10 rs1800896, ACE2 rs2285666, PEDS1 8, and IL10RB rs8178562 also appear to be genetic factors involved in the LC development [23].

Key Insights and Detailed Pathogenetic Mechanisms:

- Viral Persistence and Detection: The isolation of SARS-CoV-2 viral RNA can persist for up to 3 months in the upper respiratory tract, 2 months in serum, and 126 days in stool samples. Individual LC symptoms have been noted to last up to 20 months, indicating the long-term impact of the virus [24,25].
- Post-Hospitalization Health Outcomes: Comparisons show that patients post-COVID-19 hospitalization report their health as slightly worse than the previous year, especially when compared to patients recovering from non-COVID pneumonia. The severity of the initial infection correlates with poorer long-term outcomes and quality of life, a pattern that supports the link between acute-phase tissue damage and long-term LC symptoms [6,26,27,28].
- Age-Related Variability in LC Manifestations: Conflicting research outcomes suggest age as a factor in LC’s likelihood and severity, with various studies pointing to different susceptibilities and recovery patterns across age groups [29,30,31,32,33].
- Cognitive, Neurological Complaints, Chronic Fatigue, and Viral Activation: Evidence supports the development of numerous symptoms of chronic fatigue syndrome and other cognitive and neurological disorders in LC. These complex changes may partly relate to the activation of Epstein-Barr virus (EBV) and other herpesviruses following COVID-19 [34]. Some of the most persistent and commonly reported complaints of LC are cognitive in nature, often subjectively described as “brain fog.” These are objectively measured by deficits in executive function, working memory, attention, and information processing speed [35,36]. There is also a correlation between chronic fatigue in long COVID (LC) and latent microcirculatory disorders, which can be verified through imaging of the retinal microvascular network [37].
- Mitochondrial Dysfunction and Neuropsychiatric Effects: Elevated total mitochondrial protein levels in extracellular vesicles during acute SARS-CoV-2 infection are predictive of a high risk of LC and indicate neuropsychiatric manifestations once LC is established [38]. Additionally, LC is characterized by the prolonged presence of the SARS-CoV-2 nucleocapsid (N) protein in non-cellular vesicles [38].
- Blood–Brain Barrier Integrity and Endothelial Damage: Studies have shown that the Spike (S) protein of SARS-CoV-2 can damage the endothelium in an animal model, disrupt the integrity of an in vitro model of the blood–brain barrier (BBB), and penetrate the BBB, leading to perivascular inflammation in the CNS [39].
- Non-structural proteins of SARS-CoV-2 act as pathogenicity factors and are critical for virus survival. These proteins induce various cellular pro-inflammatory stress processes, including autophagy, oxidative and mitochondrial stress, and endoplasmic reticulum stress (ERS) [40,41,42]. Furthermore, SARS-CoV-2, including through the effects of its structural proteins S, E, and N, can induce pro-inflammatory cellular stress in various cell types via activation of the NLRP3 inflammasome [43,44,45].
- Gastrointestinal Impact and Microbiome Disruption: The long-term persistence of SARS-CoV-2 as potential bacteriophages in the intestinal microflora and the sustained disruption of the intestinal microbiome in LC have been demonstrated. Additionally, dysbiosis and intestinal barrier disruption contribute to elevated levels of lipopolysaccharides (LPSs) and other intestinal toxins, which in turn, increase pro-inflammatory status at the systemic level [46,47,48]. An increase in the relative abundance of opportunistic microorganisms in feces, accompanied by a decrease in anti-inflammatory taxa, is positively correlated with LC symptoms [49].
- Immune System Dysfunction: SARS-CoV-2-induced immune system dysfunction can potentially contribute to other acute infections or exacerbations of chronic infections, including viral infections. Moreover, LC-associated immune dysfunction may promote the reactivation of herpesviruses (CMV, EBV, HHV-6), mimicking many of the symptoms of LC [50,51,52,53,54,55].

The immune abnormalities found in LC largely fit within the paradigm of low-grade inflammation, including increased expression of inflammatory genes in monocytes, elevated serum levels of pro-inflammatory cytokines, and signs of T-lymphocyte senescence [56]. A sustained decrease in T regulatory cells (Tregs) and increased non-selective activity of innate immune cells may contribute to pathological overactivity of the immune system in LC, promoting tissue damage and autoimmune responses. Additionally, deficiencies in memory T cells, naive T cells, and antigen-presenting dendritic cells may contribute to the dysfunction of the adaptive immune response [53,57,58].

- Autoimmune Responses: SARS-CoV-2-related autoimmune processes are clinically usually latent or cofactorial to other causes of immune inflammation but, in some cases, initiate the onset of canonical autoimmune diseases [58,59,60,61,62,63]. Meanwhile, latent autoimmune processes subclinical to the development of canonical autoimmune diseases may act as pathogenetic mechanisms of LC [50,64]. Specific autoimmune response mechanisms may include molecular mimicry between SARS-CoV-2 antigens and potentially autoimmune CNS autoantigens, as well as the formation of chimeric host-virus proteins. The long-term presence of antinuclear antibodies (ANAs), antineutrophil antibodies (ANCAs), anticardiolipin (aCL), and anti-beta-2-glycoprotein-1 (anti-β2GP1) antibodies, as well as antibodies to citrullinated proteins, have been reported in LC, along with the presence of ANA and antiphospholipid autoantibodies associated with vasculitides [65,66,67,68,69].
- Several meta-analyses have demonstrated the protective role of vaccinations both before SARS-CoV-2 infection and after recovery from the disease [32,70,71,72,73,74,75,76,77,78,79]. This protection extends not only to the primary and recurrent incidence of COVID-19 but also to a reduced risk of developing long COVID (LC) [70,71,72]. This vaccine effect has been shown in elderly patients [32] as well as adolescents [73]. At the same time, contradictory data have been noted [74,75], likely due to a lack of randomized controlled trials [76]. Overall, there is a need for additional research to study the primary mechanisms of the protective effects of vaccines against LC [76,77,78].

## 3. Cellular and Tissue Stress: Relationship to Inflammation and Other General Pathological Processes

### 3.1. Summary of General Pathological Processes

Every event in the human body is governed by specific laws, including those of pathology. Any pathological process is a cascade of sequential reactions triggered in response to external or internal factors that disrupt normal physiological functions. Pathological processes display both unique and common patterns across different conditions. These patterns can be formalized as laws of pathology, which, unlike the theorems of natural sciences, are characterized by their lack of precision. The most comprehensive representations of these laws are abstract models of general pathological processes, characterized by the following features:

- Stereotypy: presence of typical features irrespective of the cause or location.
- Universality: these processes characterize the pathogenesis of many nosological entities.
- Polyetiology: the general pathological process does not depend on a single specific etiological factor.
- Self-development: the capacity to progress independently, even after the cessation of the etiological factor.
- Functionality and Dysfunctionality: Any genetically determined process is aimed at a beneficial outcome for the organism; when this is unachievable, dysfunctional systems form. These systems encompass both adaptive and pathological mechanisms. Examples include low-grade inflammation (parainflammation) and systemic hyperinflammation, whereas acute, rapidly resolving canonical inflammation typically represents a functional system.
- Phylogenetics: General pathological processes are usually not limited by evolutionary species constraints and manifest to varying degrees as general biological processes. Para-inflammatory processes, such as the encapsulation of parasites by phagocytes without the microvascular component of inflammation, are prevalent across various invertebrate phyla [80,81]. Conversely, canonical (classical) inflammation is specific to vertebrates, requiring an advanced blood microcirculation system, as well as sophisticated neuroendocrine and immune systems [82,83]. Systemic hyperinflammation associated with shock, on the other hand, seems to manifest fully only in mammals [83].
- Internal Inconsistency: pathological processes include mechanisms aimed at both maintaining and altering homeostasis, encompassing functionally divergent mechanisms that render the process manageable and balanced.
- Organizational Systematicity: As complex systems, general pathological processes are comprised of interrelated subprocesses (subsystems) and may also be components of more highly organized supersystems. These integrate various general pathological processes at the level of the whole organism, with the most comprehensive including abstract models of tumor growth regularities, canonical inflammation, and basic variants of non-classical inflammation.

### 3.2. A Brief Characterization of Canonical Inflammation and the Main Non-Classical Variants of Inflammation from the Perspective of Pathological Process Theory

Canonical inflammation is characterized by a barrier function at the inflammatory site in response to local damage, facilitated by exudative-vascular reactions and extensive leukocyte migration (Table 1). This classical inflammation, recognized since Celsus and Galen’s time, exhibits characteristic local signs: rubor (redness), calor (heat), tumor (swelling), dolor (pain), and functio laesa (loss of function). Notably, a systemic inflammatory response is not an inherent or mandatory feature of canonical inflammation; it only manifests in cases of pronounced classical inflammation, not in all instances. Its primary role is to support and enhance immune processes at the inflammation site, typically without threatening the organism’s overall health [82]. However, the chronic progression of systemic inflammatory response can manifest features similar to low-grade inflammation. To confirm systemic low-grade inflammation, it is crucial to establish its dysfunctionality, involvement of metabolic factors, and the presence of stable allostasis—a sustained change in life-sustaining homeostasis parameters.

Systemic hyperinflammation, as a special form of general pathological process, is a priori a dysfunctional process, and arises primarily from systemic microcirculatory disorders in response to significant systemic damage [84]. This may result from classical inflammation’s systemic inflammatory response dysfunction, impaired barrier function, or severe systemic alterations unrelated to the classical inflammation focus [84,85]. Common manifestations include refractory shock, resuscitation syndromes such as desensitized intravascular coagulation, secondary acute respiratory distress syndrome, and rapidly progressive multi-organ failure [85,86,87].

Low-grade inflammation (parainflammation), while typically associated with conditions like morbid obesity, metabolic syndrome, and type 2 diabetes, is also a distinct variant of general pathological processes with broader implications. This form of inflammation is not characterized by the inflammatory focus’s barrier function and can generalize within tissues and the body during chronic progression, leading to stable allostasis [88]. Low-grade inflammation arises in response to moderately damaging factors such as metabolites (meta-inflammation) [89,90], aging (inflamm-aging) [91,92], endotoxin generalization with intestinal barrier and microbiome disruption [93], and excitotoxicity [94,95]. It may underlie many somatic and neuropsychiatric disorders, including chronic stress, depression, neurodegeneration, and schizophrenia [94,95,96,97].

Features of low-grade inflammation include moderate cytokinemia, dyslipoproteinemia, insulin resistance, accelerated aging, and significant immune system compromise, with a pathogenic role for stromal macrophages, macrophage and endothelial scavenger receptors, and degenerative changes across various organs [98,99,100,101]. Unlike endotheliitis, endotheliosis, a para-inflammatory dysfunction of endothelial cells, is linked to decreased nitric oxide bioavailability due to constitutive NO synthase inhibition, leading to vasopressor hyperproduction, which may result in hypertension [102,103].

Endotheliosis, a para-inflammatory dysfunction of endothelial cells, differs from endotheliitis by involving a reduction in nitric oxide (NO) bioavailability due to the inhibition of constitutive NO synthase. This reduction leads to a hyperproduction of vasopressors such as angiotensin II (Ang-2) and endothelin I (ET-1), which can contribute to hypertension [102,103]. Conversely, endotheliitis is typically associated with the activation of inducible NO synthase and an overproduction of NO, fostering vasodilation and an exudative-vascular response at the inflammation site or in systemic hyperinflammation-induced shockogenic microcirculatory disorders [104,105]. Unlike endotheliitis, which is localized primarily in postcapillary microcirculation zones, endotheliosis spreads throughout the vascular network. This includes pathological activation, thinning of the glycocalyx, and impaired barrier function in endothelial cells of major arteries, significantly elevating the risk of atherosclerosis [106].

Markers indicative of endotheliosis or endotheliitis include elevated blood levels of glycocalyx degradation products (syndecan-1, glycosaminoglycans), activated endotheliocytes, angiopoietin-2 (Ang-2), endocan (ESM-1), ET-1, soluble forms of adhesion receptors (sICAM-1/sCD54, sVCAM/sCD106, sE- and sP-selectins/sCD62E,P), along with markers of endothelial barrier disruption such as sVE-cadherin/sCD144, vascular endothelial growth factors (VEGF), and their receptors—sVEGFR, matrix metalloproteinases (MMP), and prothrombic factors including the endothelial isoform of von Willebrand factor (vWf), soluble thrombomodulin (sTM), and PAI-1 [107].

Systemic variants of low-grade inflammation are characterized by compromised capillary network density, microvascular occlusions, and dysfunction in the contractile properties of vascular myocytes and precapillary pericytes. These changes lead to associated ischemia, sclerosis, and tissue degeneration across various organs [108,109,110]. Local morphological changes in systemic low-grade inflammation often manifest more pronouncedly in individual organs such as the kidney, liver, heart, brain, eyes, muscles, and joints, typically appearing as sclerotic and degenerative processes. These morphological changes substantially influence the pathogenesis of various diseases, including diabetic kidney disease [111], osteoarthritis [112], neurodegeneration [113,114,115], and cardiomyopathy characterized by metabolic alterations and myocardial fibrosis [116].

Distinctive features of infectious variants of low-grade inflammation separate them from endogenous types, such as meta-inflammation and inflamm-aging [117,118]. To accurately confirm systemic low-grade inflammation, mere determination of C-reactive protein levels (CRP from 3 to 10 mg/L) is insufficient; additional markers of systemic inflammatory response and endotheliosis are necessary [81]. A CRP level in this range, if accompanied by clinical phenomena but without other molecular criteria, might suggest a risk zone for low-grade inflammation.

Overall, while low-grade inflammation shares common pro-inflammatory mechanisms with canonical inflammation, there are fundamental differences in their manifestations. It is crucial to consider the potential for local transformation of parainflammation into classical inflammation, as observed in diabetic kidney disease [111]. Certain conditions, such as atherosclerosis, may exhibit characteristics of both low-grade and canonical inflammation [96]. Unlike classical inflammation, low-grade inflammation lacks a barrier function to limit the spread of alteration factors and does not lead to a microcirculatory catastrophe, as systemic hyperinflammation does. Standard outcomes across various inflammation types include tissue sclerosis, organ dysfunction, and disorders of tissue differentiation, including abnormal cell transdifferentiation, metaplasia, and malignization. The presence of both maladaptive phenomena and adaptive mechanisms in parainflammation allows organisms to survive prolonged periods under altered homeostasis (stable allostasis).

### 3.3. Cellular Proinflammatory Stress as an Elementary but Integral Unit of Pathological Processes and Tissue Stress as a Common Basis of Typical Pathological Processes

Recent studies reveal that not only pathological but also many physiological processes at the cellular level across various cell types share common molecular mechanisms associated with the development of cellular stress. This phenomenon can be termed “Cellular pro-inflammatory stress,” defined as a complex array of interconnected universal and cell population-specific processes that respond to actual or potential damaging factors [83].

Key outcomes of cellular stress include various forms of programmed cell death (apoptosis, necrosis variants), oxidative stress, transdifferentiation, malignization, and DNA-damage response. Additional responses encompass mitochondrial unfolded protein response (UPR), endoplasmic reticulum stress incorporating calcium-dependent mechanisms and UPR, autophagy, inflammasome formation, stress-induced non-coding RNAs, cytoskeletal changes, and the development of a stress-induced epigenome alongside inducible pro-inflammatory receptor and secretory phenotypes across various cell types [119]. A network of interconnected signaling pathways integrates these processes, with both extracellular and intracellular stress signals activating common and distinct signaling pathways in various cells. These pathways include collector-type protein kinases (e.g., MAPK, AKT, PI3K, PKC, ATM, ATR, AMPK, PKA, PKR, mTOR) and key universal cellular stress transcription factors, such as NF-κB, p53, AP-1, HIF, HSF, NRF2, ATF4, and STAT [119].

At the tissue, organ, and sometimes systemic levels, different types of proinflammatory excited cells are integrated through a cytokine network and other regulatory mechanisms to form the phenomenon of tissue stress [81]. Notably, various forms of tissue stress underpin not only multiple pathological processes but also many physiological processes and conditions, manifesting as physiological proinflammatory tone. This is particularly evident in barrier tissues that are in direct contact with various damaging agents, often described as being in a state of “physiological inflammation” [120]. However, from a methodological standpoint in general pathology and pathological physiology, this interpretation may be misleading. It is more accurate to discuss the physiological relevance of proinflammatory tissue stress and the presence of a proinflammatory tissue tone, rather than labeling these states as specific forms of inflammation, which are typically considered pathological processes.

**Figure 1:**
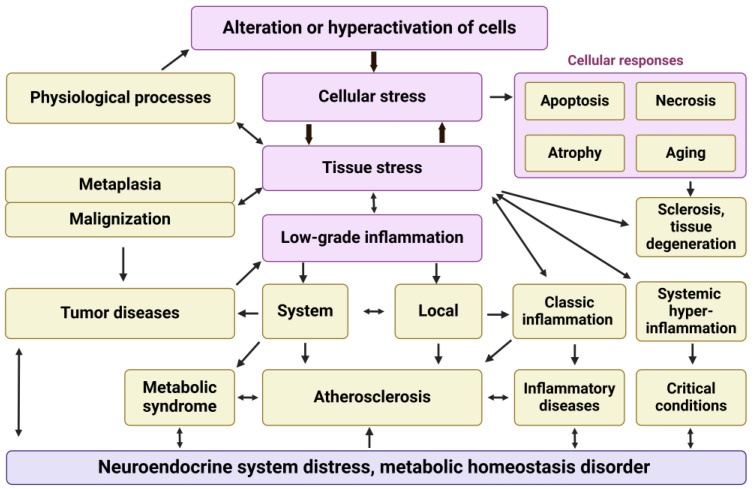
Schematic model of cell and tissue stress as a common platform for various human pathologies. Figure 1 depicts the complex interplay of cellular and physiological processes leading to various pathological conditions. The diagram is structured as follows: Alteration or Hyperactivation of Cells: This is the starting point, where cells undergo changes or become hyperactivated. Cellular Stress: Altered or hyperactivated cells experience cellular stress, leading to various cellular responses such as apoptosis, necrosis, atrophy, and aging. Tissue Stress and Low-Grade Inflammation: Cellular stress progresses to tissue stress and low-grade inflammation, which are interconnected processes. Pathological Processes: Physiological Processes: These include metaplasia and malignization, which can lead to tumor diseases. Systemic and Local Effects: Low-grade inflammation can have systemic and local effects, contributing to conditions like atherosclerosis. Classic and Systemic Inflammation: Low-grade inflammation can escalate to classic inflammation or systemic hyper-inflammation, leading to inflammatory diseases and critical conditions. Outcomes: Sclerosis and Tissue Degeneration: Resulting from cellular stress and tissue stress. Metabolic Syndrome: Arising from systemic inflammation and other related processes. Neuroendocrine System Distress and Metabolic Homeostasis Disorder: These are overarching consequences of the interactions between various pathological processes.

Consequently, the conceptual basis of various general pathological processes—as well as many physiological processes and conditions—is rooted in tissue proinflammatory stress, with cellular stress serving as its elementary but integral functional unit (Figure 1). Given this framework, it is not surprising that low-grade inflammation is pathogenetically linked to other pathological processes and underlies a vast array of specific human diseases associated with diverse etiological factors.

## 4. Low-Grade Inflammation in Long COVID and Other Viral Infections

It is now well-recognized that low-grade inflammation can be triggered by various infectious agents [121]. Viruses, in particular, contribute to this phenomenon. Despite the diversity of their molecular mechanisms, viral invasions tend to follow a limited set of typical infection scenarios, which include parasitization and a range of host responses. Notably, viral infections induce all standard processes and outcomes of cellular stress in the target cells they infect [122,123,124,125,126,127,128,129,130]. Additionally, viral infections are implicated in all major types of general pathological processes, ranging from various forms of inflammation to tumor growth, as seen with oncoviruses.

In our previous work, we described the acute phase of COVID-19 as a classical infectious-inflammatory disease, which could transition into life-threatening systemic hyperinflammation [131]. In contrast, the LC progression appears to align more closely with scenarios that involve less intense but more prolonged damaging factors, leading to persistent, torpid inflammatory responses. This chronic state reflects a shift from the acute, high-intensity responses of classical inflammation to a sustained low-grade inflammatory state, underscoring the complex interplay between viral pathogenicity and host immune responses over time.

### 4.1. Viral Infections Associated with Low-Grade Inflammation

Low-grade inflammation, particularly in the context of metabolic syndrome and type 2 diabetes, has been identified as a major risk factor for severe forms and critical complications of COVID-19 [132,133,134]. Initial endothelial glycocalyx dysfunction, which worsens as COVID-19 progresses, contributes significantly to its pathogenesis [135,136]. Moreover, endothelial dysfunction in COVID-19 may result not only from a dysfunctional immune response but also directly from the effects of the SARS-CoV-2 spike (S) protein on vascular endotheliocytes [137]. Prolonged COVID-19 infection exacerbates insulin resistance, damages pancreatic endocrine cells, and promotes chronic para-inflammatory phenomena in individuals with type 2 diabetes [138,139,140]. Additionally, a meta-analysis by Espín et al. suggests that elevated cytokine levels (IL-2, IL-4, IL-6, IL-10, IL-17, IFN-γ, CCL5, CCL3) during the acute phase of COVID-19 may predict the risk of developing LC [141]. Persistent endothelial glycocalyx damage, increased levels of circulating endotheliocytes, markers of proinflammatory vascular endothelial activation, and reduced capillary network density in various organs support ongoing endothelial dysfunction in LC [141,142,143].

Various viral infections can trigger low-grade inflammation, including:

- Post-Acute Infectious Syndromes (PAIS): This relatively new medical term refers to symptoms of long-term consequences of acute infections caused by numerous pathogenic agents, including viruses such as SARS-CoV-2 [144]. The main manifestations of PAIS encompass general poor functional status, exercise intolerance, debilitating fatigue, signs of depression, cognitive and sensory impairments, dysautonomia, musculoskeletal complaints, flu-like symptoms, as well as disturbances in gut microbiota and various immunologic dysfunctions [144,145]. Besides SARS-CoV-2, other viruses like Ebola, Dengue, Polio, SARS (SARS-CoV-1), Chikungunya, EBV, West Nile virus, and potentially alphaviruses, Ross River virus, and VZV have also been implicated in initiating PAIS [144]. Notably, PAIS following mild to moderate COVID-19 shares many characteristics with chronic diseases triggered by other pathogens, which remain poorly understood. Various causes of PAIS have been proposed, including intestinal dysbiosis, autoimmunity, microvascular endothelial damage, and long-term persistence of pathogens and their pathogenic factors [146,147,148].
- Slow Viral Diseases: These diseases manifest after a prolonged latent period with a slow, progressive course that often lasts several months to years and generally progresses until the end of life. The most well-known slow viruses include HIV and HPV [149].
- Latent Viral Infections: This concept encompasses a phase in the life cycle of certain viruses where the spread of viral particles halts following initial infection. However, the viral genome remains intact and can reactivate without reinfection, persisting indefinitely within the host. This latent stage is typical of all herpes viruses and many oncoviruses, including HIV, HPV, HBV, and Hepatitis C virus (HCV) [150,151,152,153]. The latency allows viruses to evade not only the immune system’s antiviral effects but also the protective mechanisms against cellular and tissue stress in infected tissues. Nonetheless, with prolonged persistence of viruses or their components, the distinction between the latent period and the emergence of systemic parainflammatory signs can become blurred and subtle.

### 4.2. The Relationship of Long COVID to Systemic Low-Grade Inflammation

LC is underpinned by a constellation of pathophysiological processes, including direct effects of viral persistence, reactivation of other viruses, host factors such as chronic inflammation, metabolic and endocrine dysregulation, immune dysregulation, autoimmunity, and tissue damage incurred during the acute phase of COVID-19 [154,155]. SARS-CoV-2 RNA has been noted to persist for weeks in individuals clinically recovered from COVID-19, detected in the respiratory tract, gastrointestinal tract, blood and cerebrospinal fluid [156,157], suggesting ongoing immune stimulation even without active viral replication [155]. This persistence is critical as studies have shown that while antibody titers against SARS-CoV-2 decrease over time, the T-cell response, particularly memory T cells, remains more robust [155,158]. Interestingly, a significant proportion of LC patients retain T-cell responses to SARS-CoV-2 antigens months after recovery, even when antibodies are undetectable [159]. Also, no relationship was found between antibodies against the SARS-CoV-2 nucleocapsid antigen (protein N) and clinical LC symptomatology in a studied Danish population [160]. The association between humoral and cellular responses in LC appears to be weaker than in recovered patients [161].

Meanwhile, in LC, blood levels of non-conventional monocytes (CD14^low/CD16^high) and preactivated monocytes (expressing HLA-DR), as well as activated B-lymphocytes (CD86^high/HLA-DR^high), are elevated. Additionally, T cells with increased production of IL-2 (in CD4^+/CD8^+), IL-4 (CD4^+), IL-6 (CD8^+), IFNγ, and IL-17 (CD4^+), as well as TNF-α (CD8^+), are noted, but the level of CD4^+ central memory T cells is decreased [162]. Moreover, increased T-lymphocyte senescence and monocyte activation have correlated with LC severity [56]. A systematic review by Haunhorst et al. indicated that a decrease in blood Treg counts induced by SARS-CoV-2 infection can persist for several months [163]. Although the limited number of studies on Treg in LC has not allowed for definitive conclusions regarding their role in the etiology of LC, significantly altered proportions of Treg in the CD4^+ T-cell population have been observed almost a year after infection, suggesting a possible involvement of Treg in the pathogenesis of an uncoordinated immune response to SARS-CoV-2 in LC [163,164].

In summary, SARS-CoV-2 infection causes a prolonged enhancement of the pro-inflammatory transcriptional status in many immunocytes, which may predispose patients in the post-acute period to the development of long-term health consequences, including autoimmune diseases, reactivation of other viruses, and disruption of the host’s immune system and microbiome ecosystem [165]. Simultaneously, LC may manifest as dysfunction of the antimicrobial mechanisms of innate and adaptive immunity, potentially contributing to secondary infections and the reactivation of latent chronic infections [27,166].

A more reliable sign of low-grade inflammation is moderate manifestations of a systemic inflammatory response in the absence of classical inflammation. In a systematic review by Lai Y.J. et al., key immunologic markers in blood (plasma/serum) of different LC variants were identified, which included elevated levels of IL-6, TNF-α, IFN-α, IFN-γ, GM-CSF, M-CSF, CCL2, CCL3, CXCL10, Ang-2, and matrix metalloproteinases (MMP-1, MMP-9); acute-phase proteins (CRP, fibrinogen, and ferritin); and markers of endotheliosis: VEGF, sVEGFR, sVCAM-1, ET-1, PDGF-B, sTM; and a paracoagulation marker (D-dimer) [167]. Additionally, other criteria of endotheliosis observed in patients with LC symptoms include elevated plasma levels of sVE-cadherin, sE-selectin, sICAM-1, vWf [107], as well as other signs of systemic parainflammation, including decreased NO bioavailability [168], elevations in blood and other cytokines (IL-1β, IL-2, IL-10) [15,169,170], and in some patients, signs of thrombophilia and systemic mast cell activation [15,166,171]. At the same time, the criteria of low-grade inflammation (CRP 3-10 mg/L, increased fibrinogen levels) as well as an increased neutrophil/lymphocyte ratio are detected in LC, even after a mild course of COVID-19 [172]. Proteomic studies in individuals with LC show both the presence of common signs of a systemic inflammatory response, an increase in serum/plasma proinflammatory cytokines, chemokines, soluble forms of cytokine receptors, and activation of transcription factor NF-κB in leukocytes, but also the division of these patients into two groups: one dominated by elevated interferon-γ (IFN type II), while the second shows signs of persistent neutrophil activation, likely caused by IFN type I [173].

### 4.3. The Association of HIV and Herpesviruses with Systemic Low-Grade Inflammation

Low-grade inflammation as a general pathologic process is polyetiologic. It is therefore unsurprising that signs of systemic low-grade inflammation are detected in various viral infections, primarily categorized under Post-Acute Infectious Syndromes (PAISs), “slow virus” diseases, and chronic sluggish viral infections. We focus here on three characteristic examples: (1) HIV infection, widely recognized as one of the most well-studied “slow viruses”; (2) herpesviruses (CMV, EBV, HHV-6), implicated in the pathogenesis of LC; and (3) HCV infection, which exemplifies a chronic infection with significant systemic manifestations.

HIV: Despite effective antiretroviral therapy, HIV-infected patients exhibit persistent low-grade inflammation and chronic immune activation, associated with an increased risk of cardiovascular disease, osteoporosis, anemia, and low-intensity endotoxemia, among other conditions not directly related to AIDS [174,175]. Notable in HIV infection are accelerated aging and metabolic changes typical of low-grade inflammation, such as insulin resistance and lipotoxicity, with dysfunctional pro-inflammatory lipokines [176,177]. Guerville et al. found elevated blood levels of IL-1β and higher average values of IL-18 and systemic oxidative stress markers in a subgroup of HIV-infected patients [178]. Further, elevated levels of IL-6, CRP, sICAM, ET-1, D-dimer, and soluble scavenger receptors (sCD14, sCD163) have been observed [179,180,181,182,183].

Herpesviruses: Reactivation of latent herpesviruses, evidenced by higher antibody titers, typically occurs when cell-mediated immunity is compromised. Increased levels of IL-6 and CRP during simultaneous reactivation of CMV and EBV are noted, particularly in the context of neuropsychiatric stress and, occasionally, depression [184]. It is important to recognize that psychoemotional stress and depression can lead to immune dysfunction and further exacerbate low-grade inflammation [95]. Additionally, patients with the highest CMV antibody titers exhibit an increased mortality risk from cardiovascular diseases and all-cause mortality, a relationship partly explained by moderately elevated levels of IL-6 and TNF-α [185]. EBV reactivation can stimulate human monocytes and vascular macrophages to produce TNF-α and IL-6 and enhance the adhesive properties of endothelial cells through the inducible expression of VCAM-1 and ICAM-1 [186]. Furthermore, psychological stress, particularly noted in older adults, has been identified as a potential trigger for CMV reactivation, linking stress to immunity, oxidative/antioxidant balance, and aging [187]. However, another study found no correlation between CMV antibodies and the progression of inflamm-aging marked by CRP and TNF-α levels [188]. It is necessary to consider that not all patients in this category will develop signs of systemic low-grade inflammation, and the systemic inflammatory response in low-grade inflammation is not always pronounced; verifying this response objectively requires comprehensive criteria.

Regionally confined HHV-6 reactivation can cause immune dysfunction, neuroinflammation, neurodegeneration, major depressive disorder, and ME/CFS [189,190,191,192,193], potentially accounting for many localized LC phenomena. However, assessing the impact of HHV-6 on systemic low-grade inflammation processes may still be premature. Cases of life-threatening systemic hyperinflammation (viral sepsis) linked to disseminated herpes simplex virus 1 and 2 (HSV-1, HSV-2) infections have been documented [194,195]. HSVs may also play roles in systemic and local para-inflammatory processes, including neuroinflammation and neurodegeneration [196].

The role of herpesviruses in systemic low-grade inflammation is likely more significant than currently acknowledged. However, distinguishing the effects of herpesviruses from other infectious and non-infectious risk factors of systemic and local parainflammation necessitates the use of advanced methodologies, including Mendelian randomization [197,198].

Hepatitis C Virus (HCV): In viral hepatitis C, extrahepatic manifestations that involve various organ systems and symptoms of systemic parainflammation are common [199]. These manifestations often include insulin resistance and systemic autoimmune phenomena such as circulating immune complexes, cryoglobulinemia, and associated vasculitis, contributing to chronic systemic low-grade inflammation and affecting the development of extrahepatic manifestations, particularly cardiovascular diseases [199,200].

In conclusion, the development of low-grade inflammation is a common pattern across many viral infections, intimately linked to both systemic and local (organ-specific) phenomena, including in conditions such as LC.

## 5. Local Phenomena of Low-Grade Inflammation in LC and Other Viral Infections

The foundation of pathokinesis in low-grade inflammation involves an increase in physiological pro-inflammatory tissue stress, leading to allostasis (stable changes in a number of homeostasis parameters) without the characteristic signs of canonical inflammation. In this context, we can discuss local phenomena of low-grade inflammation in relation to individual organs and tissues. It is important to consider that local phenomena of low-grade inflammation during viral infections can precede systemic parainflammation, especially in relatively young individuals who do not have chronic diseases.

### 5.1. Possible Role of Low-Grade Inflammation in CNS Dysfunction in LC and Other Viral Infections

Currently, functional changes in the CNS are prominent in LC symptomatology. The role of microglial activation linked to viral infections in the development of mild neuroinflammation (neuroparainflammation) is undeniable, not only in the pathogenesis of neurodegenerative diseases and accelerated brain aging [201,202,203] but also in canonical neuropsychiatric diseases such as schizophrenia and depression [204,205,206]. Moreover, the molecular mechanisms of cellular and tissue proinflammatory stress manifest not only in pathologies but also support many physiological processes of the CNS. Thus, understanding the physiological functions of these mechanisms is essential for assessing their pathogenetic significance.

#### 5.1.1. The Physiological Role of Cellular and Tissue Stress in the Central Nervous System and the Path of Its Pathological Transformation

The brain, composed of highly differentiated tissues with limited regenerative capacity for neurons, akin to postmitotic cells, is particularly susceptible to sclerosis and neurodegenerative changes. This susceptibility is evidenced by the frequent detection of neurodegenerative areas in the brains of individuals who do not exhibit symptoms of canonical central nervous system diseases, as revealed through MRI [207,208]. However, only a subset of these individuals will progress to canonical neurodegenerative diseases such as Alzheimer’s or Parkinson’s disease. Given its vulnerability, even moderate changes in key homeostasis parameters can trigger apoptosis and various forms of programmed cell death (e.g., pyroptosis, necroptosis) in neurons.

**Figure 2:**
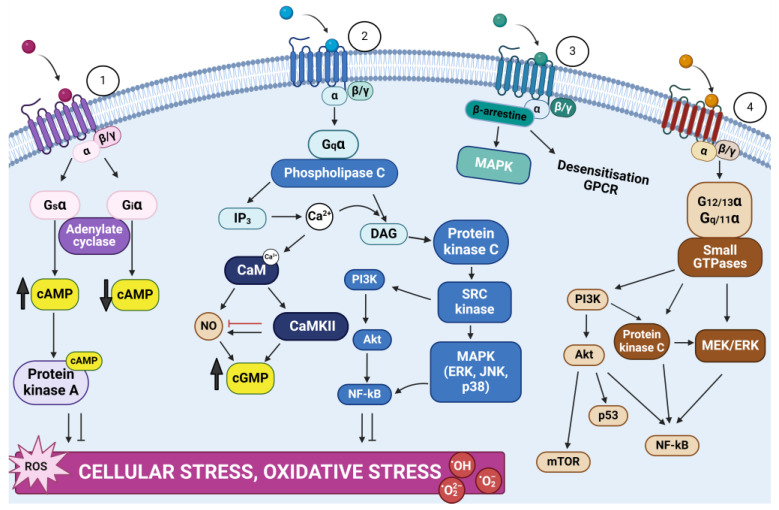
The role of GPCR receptors in cellular stress development [95].

Despite the CNS’s inherent protections—such as barriers, immune privilege, non-reliance on potentially lipotoxic fatty acids for bioenergetics, and limited microglial activity akin to stromal macrophages—these defenses do not completely prevent damage [95]. Moreover, the CNS’s high metabolic rate and intensive transmembrane cation transport necessitate robust protective mechanisms to maintain physiological pro-inflammatory tone under both normal and extreme physiological conditions. Notably, almost all types of neurotransmitters acting through metabotropic G-protein-coupled receptors (GPCRs) can stimulate cellular stress signaling pathways in neurons (Figure 2) [95].

Under physiological conditions, the role of classical inflammation and immunity mediators in maintaining the viability of neurons and glial cells is subtle yet significant, mediated through cytokine receptors that primarily act via non-receptor tyrosine kinases, such as Janus kinase (JAK) [200]. In neuroparainflammation, the pathogenetic influence of JAK and other cytokine-dependent mechanisms intensifies, particularly in promoting the polarization of microglia towards the pro-inflammatory M1 phenotype [201]. These pathological changes can be triggered by both non-infectious factors, such as chronic cognitive stress, and various infectious agents, including viral infections.

This figure illustrates the intricate mechanisms through which GPCRs contribute to cellular stress. Upon ligand binding, GPCRs interact with trimeric G proteins (αβγ), leading to GDP-GTP exchange on the Gα subunit. This exchange results in the dissociation and activation of the Gα subunit. The cycle completes with GTP hydrolysis and the reassociation of the Gα subunit with Gβγ, thus inactivating the G protein. G proteins are categorized into four families—Gs, Gi, Gq, and G12/13—each triggering distinct signaling pathways. Gs activates adenylate cyclase, increasing cAMP levels and PKA activity, while Gi inhibits this process. Gq stimulates PLC, generating IP3 and DAG, which release Ca^2+^ and activate PKC. This cascade influences various stress pathways, including PKC/MEK/ERK, PKC/Sc/MAPK, and PKC/Sc/PI3K/AKT. PI3K signaling, crucial for cell stress responses, can be activated by Gα/PKC, Gα/small GTPases, and Gβγ, impacting cell cycle, proliferation, and apoptosis. Adenylate cyclase, modulated by Gs or Gi, produces cAMP that activates PKA, generally exerting anti-inflammatory effects. However, the specific impact of cAMP on cellular stress is complex. Calcium, calmodulin, and CaM kinases also play significant roles, affecting NO production and cGMP formation. Additionally, small GTPases (e.g., Ras, Rho, Rab) activated by G12/13 and Gq modulate signaling through PI3K, ERK, and AKT.

Finally, GPCRs can activate MAPK via β-arrestin, which also mediates GPCR desensitization and internalization, highlighting the complex regulatory network of GPCRs in cellular stress mechanisms.

Specifically in LC, morphofunctional changes within the limbic system are evident, manifesting as psychosomatic, psychoemotional, cognitive, and other clinically significant dysfunctions [209,210,211,212,213,214,215]. The development of neurodegenerative zones in the cortical and subcortical limbic areas highlights the role of neuroinflammation in the pathogenesis of these conditions.

**Figure 3:**
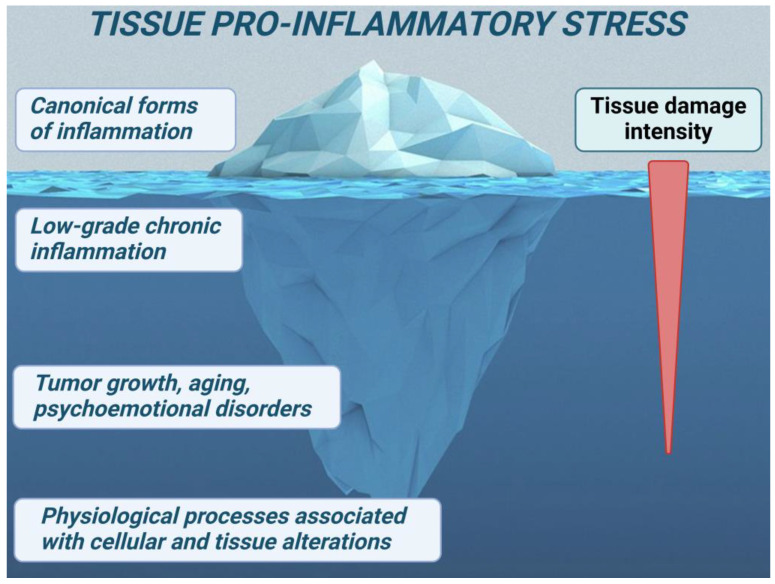
The integrative role of tissue stress (the “Iceberg” model) in various pathological and extreme physiological processes.

Thus, depending on their intensity, duration, and context, the molecular mechanisms of cellular proinflammatory stress contribute to both physiological processes and various pathological states. Among these, the processes of canonical (classical) inflammation represent just the visible tip of a vast ‘pathogenetic iceberg’ (Figure 3).

#### 5.1.2. Myalgic Encephalomyelitis/Chronic Fatigue Syndrome (ME/CFS)

ME/CFS, also known as Systemic Exertion Intolerance Disease (SEID), represents one of the most severe dysfunctions involving the limbic-reticular complex and its connections with the frontal cortex and other neocortical areas [216,217,218,219]. Although ME/CFS and similar conditions are not the most typical manifestations, they are among the most concerning for public health due to the profound chronic fatigue and intolerance to physical and psycho-emotional stress that often lead to disability in affected individuals.

The etiology and pathogenesis of ME/CFS remain largely unclear. However, increasing evidence suggests that ME/CFS is not merely a neuropsychiatric disorder but also involves significant contributions from the immune system and low-grade neuroinflammation (neuroparainflammation) [219,220]. In 2015, the Committee on Diagnostic Criteria for ME/CFS redefined the disorder as SEID and specified diagnostic criteria [221], which include:

1. A significant reduction in the ability to engage in pre-illness levels of activity that persists for more than six months, accompanied by fatigue.
2. Post-exertional malaise, where physical or cognitive exertions trigger a worsening of symptoms.
3. Unrefreshing sleep, alongside either cognitive impairment or orthostatic intolerance.

It is noteworthy that many symptoms of ME/CFS/SEID are similar to those observed in post-acute infection syndromes (PAIS), including LC. Diagnosing ME/CFS is challenging because numerous chronic inflammatory diseases can mimic its symptoms, particularly central nervous system dysfunctions presenting with individual SEID symptoms. Some symptoms of ME/CFS/SEID, such as asthenia and psychasthenia, along with fever and activation of the hypothalamic-pituitary-adrenal axis, are typical signs of a systemic inflammatory response [81]. ME/CFS is thus a diagnosis of exclusion.

Importantly, ME/CFS can be caused by localized low-grade inflammation in specific parts of the central nervous system, including latent chronic viral infections and post-viral conditions. Enteroviruses and herpesviruses, such as EBV, HHV-6, and CMV, are considered potential etiological factors for viral ME/CFS [46,205,206]. Extensive evidence now links ME/CFS to LC [222,223,224,225,226,227,228].

Proposed pathogenetic mechanisms for ME/CFS in viral infections include virus persistence in CNS cells leading to local neuroinflammation, development of autoimmune and secondary autoinflammatory responses targeting the CNS, disruption of the blood–brain barrier, and dysfunction in the interplay between the immune and autonomic nervous systems. Furthermore, local circulatory disorders in the CNS have been identified [209,228,229,230,231,232]. Additionally, administration of the SARS-CoV-2 spike protein into the brains of mice has shown delayed effects on cognitive function, mirroring LC symptoms including neuroinflammation and microgliosis in the hippocampus, with notable involvement of Toll-like receptor 4 (TLR4), particularly in microglial cells [233]. This finding suggests long-term effects of the spike protein on the brain endothelium, potentially disrupting the blood–brain barrier [38].

Evidence also suggests that prior SARS-CoV-2 infection may facilitate the emergence or acceleration of canonical neurodegenerative diseases [234,235,236,237]. Research indicates that tau protein accumulation, a hallmark of neurodegenerative diseases and to a lesser extent in LC, is characteristic [238,239]. Therefore, further research is essential to definitively determine the role of virus-induced neuroparainflammation in the pathogenesis of ME/CFS, particularly in the context of its relationship with LC.

#### 5.1.3. Long COVID and PTSD

As was already mentioned, between 31% and 69% of COVID-19 patients experience post-acute sequelae of SARS-CoV-2 infection (LC), indicating that a significant portion of survivors suffer long-term effects. The prevalence remains consistently high over time, with about 54% of patients experiencing symptoms at various stages after infection: 1 month, 2–5 months, and 6 months or more [8,9,13,14,28,29,30,31,32,33,34,35,36,240]. In particular, gender differences in the risk of PTSD, where women are more susceptible than men, may suggest similar gender-specific risks in LC, given its overlapping symptomatology with chronic stress conditions [241].

Several studies highlight a significant decrease in cortisol levels in patients with LC, which is not associated with damage to the adrenal glands, suggesting dysregulation of the HPA axis rather than organic adrenal pathology [162,241,242,243]. However, certain authors have proposed the involvement of the pituitary gland in post-COVID-19 syndrome [53,244]. This is because the ACE2 receptor, which facilitates the entry of SARS-CoV-2 into cells, is expressed within the hypothalamo-pituitary axis [244].

Decreased cortisol levels are significant predictors of the LC status, achieving high diagnostic accuracy (AUC of 0.96) in models adjusted for demographics and other factors [162]. Plasma cortisol levels were significantly lower in participants who reported respiratory symptoms three months after infection, accompanied by higher titers of interferon autoantibodies [245]. Furthermore, hypocortisolemia and hypoactivation of the HPA axis reported in LC are also reminiscent of what was previously described in 2005 in the post-SARS ‘sickness syndrome’ type condition characterized by symptoms such as malaise, anorexia, fatigue, and myalgias [246]. Improvement in cognitive functioning correlates with increased cortisol levels [247]. Furthermore, plasma cortisol levels have a significant negative correlation with fatigue scores and a positive correlation with quality of life [248].

The hypoactivation of the HPA axis observed in LC mirrors the altered cortisol profiles seen in PTSD, indicating similar physiological responses to chronic stress and immune dysregulation. Importantly, neither LC nor PTSD affects all individuals exposed to initial triggering events (SARS-CoV-2 infection and trauma, respectively). This selective impact suggests specific vulnerability factors in certain populations. Additionally, both conditions exhibit a notable gender disparity, disproportionately affecting women. In PTSD, prevalence rates differ significantly between different groups, with women having a two to three times higher risk of developing PTSD compared to men [241]. This gender-specific susceptibility is also mirrored in LC, where similar patterns of risk and severity have been observed, reinforcing the parallel between these conditions. The prevalence of PTSD varies with point prevalence ranging from 1.2% to 87.5% in military populations, and lifetime prevalence rates from 7.7% to 17.0%, emphasizing the variability and impact of traumatic experiences across different populations [249,250]. Such parallels enhance our understanding of potential common mechanisms underlying these conditions and could guide targeted interventions and support for affected groups. Importantly, PTSD is one of the complications of chronic mental stress, which in turn is linked to low-grade inflammation [95,201], potentially explaining the overlap of many neurogenic symptoms between LC and PTSD.

### 5.2. The Problem of Arthritis and Long-Term Arthralgia Associated with Long COVID

It is estimated that approximately 1% of all cases of acute arthritis, with a myriad of pathogens involved, can be traced back to a viral etiology. Known viruses include Parvovirus B19, HBV, HCV, HIV, Alphaviruses, HTLV-1, EBV, CMV, CHIKV, Flavivirus, and RuV [250,251]. These viruses may provoke joint symptoms through various mechanisms: direct viral invasion of the joint, formation of immune complexes, and immune modulation that drives chronic inflammation. The synovial lining, rich in blood vessels and nerves, is particularly susceptible to hosting arthritis-inducing viruses, which can perpetuate the inflammatory response by recruiting professional inflammatory cells to the joints [251].

Often, viral infections can transiently trigger latent autoimmune processes, leading to the production of low titers of autoantibodies, such as rheumatoid factor and antinuclear antibodies [252]. Unlike immune-mediated rheumatologic diseases, virally mediated arthritis tends to be self-limiting. Typically, viral arthropathy presents not as classical joint inflammation but as transient arthralgia. This form of arthralgia is primarily due to a low-intensity inflammatory process in the synovial membrane of the mobile joints [253]. This parainflammatory process can extend into the hyaline cartilage of mobile joints, where classical inflammation is impeded by the absence of blood vessels and innervation.

Arthralgia is a hallmark symptom in both acute COVID-19 and LC [254,255,256]. There is substantial evidence indicating the emergence of canonical rheumatic diseases closely linked temporally with COVID-19, such as rheumatoid arthritis, polymyalgia rheumatica, reactive arthritis, axial spondyloarthritis, and systemic connective tissue diseases [255,256,257,258]. Moreover, long-term arthralgia, potentially stemming from latent viral synovitis and localized low-grade inflammation, may accelerate the aging and degeneration of hyaline cartilage and the underlying bone structure of the joint. This process, coupled with inflamm-aging, constitutes a risk factor for the onset and progression of osteoarthritis [259,260,261].

### 5.3. Osteomyopathy as an LC Phenomenon

Epidemiological data indicate that myalgia, muscle and joint dysfunction, and bone fragility are prevalent consequences in patients with COVID-19. In this context, it is suggested that SARS-CoV-2 infection accelerates aging effects on the musculoskeletal system, exacerbating conditions like osteosarcopenia [262]. A frequent non-pulmonary manifestation of acute COVID-19 is skeletal muscle myopathy, which can vary from mild myalgia to myositis, or even rhabdomyolysis in severe cases [263]. Although direct infection of myocytes has not been conclusively demonstrated, the inflammatory myopathies associated with COVID-19 are thought to be immune-mediated [264]. Myalgia and muscle weakness can persist for a year or more post-infection [265,266], and are observable even after mild acute COVID-19 episodes [267]. Additionally, mitochondrial alterations, capillary damage, and signs of latent inflammation may remain evident in muscle biopsies for extended periods [265,268]. In some cases, data suggest that muscle weakness and pathological fatigue in LC may involve dysregulation of the autonomic system, focusing on cholinergic and sympathetic efferent activity, even in the absence of overt myopathic changes [269].

SARS-CoV-2, via ACE2 receptors, can infect bone cells, including osteoblasts and osteoclasts. The involvement of low-grade inflammation in bone tissue and various pro-inflammatory T-helper cells (Th1, Th17), macrophages (M1, M2b), and their cytokines (IL-17, TNF-α, IFN-γ, IL-6, etc.) can reduce the mineral density of ostensibly healthy bone tissue [270,271]. The disruption of bone remodeling triggered by SARS-CoV-2 may lead to osteoporosis, whose effects can linger well beyond the acute phase of COVID-19 [272]. Moreover, the long-term use of glucocorticoids during acute COVID-19 and disturbances in the intestinal microbiome may exacerbate this pathological process [271]. To date, Mendelian randomization has not confirmed a direct link between osteoporosis and the severity of acute COVID-19 in European populations [273]; however, the risk of osteoporosis in LC might be significantly linked to even mild cases of COVID-19.

### 5.4. Kidney Pathology in LC

While kidney disease symptoms are not typically associated with LC, clinical evidence suggests that LC complications require nephrological monitoring. SARS-CoV-2 can directly infect renal cells, including tubular epithelium as well as glomerular endotheliocytes and podocytes, potentially causing glomerular damage followed by fibrosis [274]. This direct cellular damage by the virus occurs independently of the immune system [275]. Such findings help explain both acute kidney injury in COVID-19 patients and the development of chronic kidney disease in LC [276]. Additionally, subclinical inflammation (parainflammation) may persist for months, leading to a gradual decline in kidney function and, in some instances, chronic renal failure [277]. Biochemical alterations, such as disruptions in creatine phosphate metabolism, unutilized glomerular filtrate, and the tryptophan paradigm associated with COVID-19-specific damage to proximal tubular cells, may also contribute to renal dysfunction in LC [278]. It has been observed that the N protein of SARS-CoV-2 persists in the renal tubule epithelium of recovered COVID-19 patients. The presence of the N protein, even without signs of viral replication, may be significant for the recurrence of kidney disease and have lasting effects on the immune system [279].

### 5.5. Liver Pathology in LC

The long-term effects of COVID-19 on liver function remain elusive, but some studies report a persistent elevation of blood enzymes indicative of hepatocyte damage, alongside increased ferritin levels [280]. Liver biopsies from COVID-19 patients often reveal non-specific morphological changes in the liver parenchyma, which are likely results of indirect damage from the viral infection or therapeutic interventions [281]. In the review article by Backer et al., it was noted that post-COVID-19 changes such as Kupffer cell hypertrophy in sinusoidal capillaries, moderate steatosis, elevated levels of atherogenic lipoproteins, and alterations in the intestinal microbiome might be risk factors for Metabolic Dysfunction-Associated Steatotic Liver Disease (MASLD) [282]. Additionally, several severe cases of post-COVID-19 cholangiopathy have been documented, suggesting potential for long-term liver disease [283]. Overall, post-COVID-19 hepatic alterations do not fit the classical picture of hepatitis but may be linked, at least in part, to local low-grade inflammation. Nonetheless, these liver changes are not unique to LC, as prolonged hepatitis-like symptoms have also been observed in other viral infections, including HIV and herpesviruses (EBV, CMV, HHV-6) [284].

### 5.6. Cardiovascular Pathology in LC 

Systematic reviews and meta-analyses indicate that past COVID-19 infection accelerates the progression of coronary atherosclerosis, local cardiac fibrosis, and enhances the risk of cardiac arrhythmias, heart failure, myocardial infarction, and cardiogenic shock [285,286,287,288,289,290,291,292,293]. The likelihood of developing these complications is also influenced by patient age and the presence of comorbid chronic diseases [288]. Notably, an increased risk of these complications has been observed even in individuals who were not hospitalized for acute COVID-19 [288]. Autoimmune reactions and sympathetic hyperactivation are posited to contribute to the cardiovascular manifestations of LC [290]. Additionally, prior COVID-19 exposure heightens the risk for relatively rare autoimmune vasculitides, as supported by several systematic reviews [291,292,293].

### 5.7. Pathology of the Respiratory System in LC

Respiratory symptoms frequently reported in LC include shortness of breath, cough, and chest pain or tightness. A meta-analysis indicates that three months post-COVID-19, shortness of breath affects 32% of patients, while the prevalence of cough is estimated at 8.6%, and chest pain at 6.6% [294]. These symptoms may correlate with the exacerbation of chronic bronchopulmonary diseases and regulatory dysfunctions of the central nervous system. However, currently, there is no clear evidence to link these respiratory changes directly to local low-grade inflammatory phenomena within the respiratory organs.

## 6. NSAIDs and Their Role in Subclinical Inflammation and Long COVID

Nonsteroidal anti-inflammatory drugs (NSAIDs) have been proposed as a strategy to counteract low-grade inflammatory status and prevent age-related inflammatory diseases [295]. Epidemiological data suggest that long-term NSAID use might reduce the risk of developing Alzheimer’s disease (AD) by attenuating pro-inflammatory prostanoid synthesis [295,296]. This aligns with findings that NSAID use lowers the prevalence of AD, although clinical trials have not demonstrated an effect on disease progression [296,297,298,299,300,301].

The discrepancy between epidemiological and clinical trial findings may be due to the therapeutic window of NSAIDs being pre-symptomatic or the presence of healthy user bias [302]. Healthy user bias occurs when healthier individuals are more likely to use therapies, leading to misleading associations between therapy use and reduced disease prevalence. Analyses show similar reductions in Alzheimer’s disease diagnoses among users of various pain-relievers, such as aspirin, celecoxib, and paracetamol, despite differences in their mechanisms [302]. Another possibility is that certain NSAIDs (e.g., ibuprofen, indomethacin, sulindac) reduce the production of the β-amyloid peptide independently of cyclo-oxygenase activity [303].

Regarding long COVID (LC), anti-inflammatory compounds show potential [304]. The inflammatory component in the pathogenesis of COVID-19 and LC suggests the use of anti-inflammatory therapy. Evidence suggests NSAIDs can effectively manage various forms of low-grade chronic inflammation, such as meta-inflammation and inflamm-aging [305,306,307,308,309,310,311].

However, the use of NSAIDs for COVID-19 treatment remains controversial. Early in the pandemic, concerns were raised about the potential for NSAIDs to worsen COVID-19 symptoms, but subsequent reviews and studies did not find conclusive evidence linking NSAIDs to worsened outcomes in COVID-19 patients [312,313,314]. Achieving an optimal balance between protective and adverse effects is crucial [315].

Milder anti-inflammatory agents than NSAIDs, such as antioxidants and natural modulators of cellular and tissue stress, may be promising for treating LC. Anti-inflammatory natural products also show potential in managing chronic inflammatory conditions by acting on various molecular targets relevant to the inflammatory response [316]. Of the ongoing RCTs for treating long COVID, only rintatolimod and LYT-100 (deupirfenidone), which are not NSAIDs, have shown modest to high potential [317]. Several studies indicate that Rebamipide, an amino acid derivative, has mucosal protective actions and reduces intestinal permeability, thereby affecting the inflammatory status [318]. The limited success of most drug candidates highlights the complexity of treating long COVID, and treatment may need to be tailored to specific subtypes [317].

Overall, the role of NSAIDs and other anti-inflammatory agents in managing long COVID remains under investigation, with mixed outcomes. Tailoring treatments to specific subtypes of long COVID and using integrative approaches with natural anti-inflammatory compounds could provide additional benefits. Further research is essential to determine optimal strategies for managing chronic inflammatory conditions and long COVID.

## 7. Conclusions

LC represents a multisystem pathology characterized by a diverse array of clinical symptoms and pathogenetic phenomena. The origins of LC are multifactorial and only partially attributed to the prolonged persistence of SARS-CoV-2 and its antigens. Other potential contributors include low-intensity autoimmune reactions, pathological hyperfunctions of innate immunity, diminished protective functions of adaptive immunity, reactivation of latent chronic viral infections, as well as the long-term effects of tissue damage during the acute phase of COVID-19, dysbiosis, and impaired intestinal barrier function. To effectively systematize and classify the varied and often contradictory phenomena of LC, it is prudent to approach its pathogenesis from the perspective of general pathology. This involves identifying which models of general pathological processes predominantly represent the main patterns of LC pathogenesis. Unlike acute COVID-19, the primary phenomena of LC do not conform to the models of canonical inflammation or life-threatening systemic hyperinflammation but align more closely with patterns typical of low-grade inflammation or systemic and local parainflammation. This classification is not exclusive to coronavirus infections but is also characteristic of other post-acute infectious syndromes and chronic viral infections. This understanding supports the application of methodological approaches from both personalized medicine and standard protocols for prevention, diagnosis, and treatment of pathogenetically similar LC-like diseases.

Meanwhile, it is necessary to consider that low-grade inflammation in the complex pathogenetic system may be interrelated with various forms of canonical inflammation of infectious, autoimmune, allergic, and other natures. These forms share common mechanisms of pro-inflammatory tissue and cellular stress but also have their own unique characteristics. From this perspective, low-grade inflammation may not represent a specific disease or syndrome, but rather a multifaceted complex of different clinical definitions.

## 8. Materials and Methods

### 8.1. General Characterization of the Study

This paper is structured as a scoping or conceptual review. It does not employ a meta-analytical approach based on Cochrane criteria since it does not primarily address specific clinical or pharmacological questions. Instead, references to systematic reviews and Mendelian randomizations were prioritized to analyze clinical patterns. The study also incorporates selected heuristic analysis methods typical of narrative reviews, along with systems analysis and other methodological approaches of general pathology.

The literature was sourced from databases such as PubMed, PMC, MedLine, Google Scholar, and Science Direct. Search terms included: “Long COVID” and its synonyms, “SARS-CoV-2”, “persistence”, proteins (“S”, “M”, “N”, “E”), “central nervous system”, “ME/CFS”, “limbic system”, “blood-brain barrier”, “pituitary-hypothalamic-adrenal axis”, “cardiovascular system”, “endothelium”, “atherosclerosis”, “microcirculatory disorders”, “respiratory system”, “digestive system”, “microbiome”, “liver”, “musculoskeletal system”, “arthralgias”, “osteoarthritis”, “immune system”, “autoimmunity”, “cytokines”, “low-grade inflammation”, “inflamm-aging”, “meta-inflammation”, “metabolic syndrome”, “diabetes”, “obesity”, and “lipokines”.

### 8.2. Principles of Inclusion and Exclusion

Priority was given to review articles and original publications in English from the past ten years, particularly those with high citation rates. We aimed to exclude redundant publications or those detailing specific mechanisms that did not significantly enhance the understanding of the broader aspects of LC. Publications not available in full were largely excluded, as were those that could not substantially contribute to a comprehensive understanding of the topic.

For foundational concepts such as the general theory of pathological processes, classifications of inflammation, and mechanisms of cellular and tissue stress, we relied extensively on our own published work. While these references provide a systematic and detailed perspective on current issues in general pathology, they may also introduce a degree of subjective bias.

### 8.3. Operational Limitations

Despite a systematic approach to selection of the literature, the potential for selection bias remains a limitation, as relevant studies might have been inadvertently omitted due to specific search criteria or database limitations. The rapidly evolving nature of LC research means that new findings could emerge shortly after this review’s publication, potentially altering the current understanding of its pathogenesis. The focus on English-language studies may restrict the review’s comprehensiveness, as significant studies in other languages might have been overlooked, introducing a potential bias.

Furthermore, despite efforts to maintain objectivity, the inherent nature of conceptual reviews poses a risk of subjective bias in the presentation, analysis, and interpretation of data. Another limitation is the underappreciation among many clinicians of the significance of general pathological processes as a fundamental category in medicine.

Interventionary studies involving animals or humans, and other studies that require ethical approval, must list the authority that provided approval and the corresponding ethical approval code.

Finally, our study focused on the issue of low-grade inflammation as a general pathological process in LC. However, we did not cover various phenomena of canonical inflammation related to LC, which can manifest in different organs and tissues of patients suffering from LC. These issues have been addressed in review articles by other authors, including [285,319,320,321,322]. Another aspect of canonical inflammation in LC, which was not included in the aims and objectives of our study, is considering LC as a risk factor for the onset of various inflammatory diseases or the progression of existing inflammatory diseases [323,324,325,326,327]. This primarily concerns immune-dependent diseases, whose association has been established, namely various allergic, autoimmune, and infectious diseases, but also should be taken into account in other pathological conditions as well [237,238,239,240,241,242,243,244,245,246,247,248,249,250,251,252,253,254,255,256,257,258,259,260,261,262,263,264,265,266,267,268,269,270,271,272,273,274,275,276,277,278,279,280,281,282,283,284,285,286,287,288,289,290,291,292,293,294,295,296,297,298,299,300,301,302,303,304,305,306,307,308,309,310,311,312,313,314,315,316,317,318,319,320,321,322,323].

## Figures and Tables

**Table 1:** Typical characteristics of various types of inflammation.

Inflammation Type	Definition	Mechanisms and Agents	Primary Role	Key Features	Refs
Canonical Inflammation	A classic inflammatory response characterized by local signs and a barrier function.	- Response to local damage - Exudative-vascular reactions - Leukocyte migration - Triggered by local damage. The systemic inflammatory response is primarily aimed at providing resources to the site of inflammation.	Supports immune processes at the site of inflammation without systemic threat. Localizes and eliminates the damaging factor. Ends with tissue regeneration or repair (sclerosis)	- Local signs: redness, heat, swelling, pain, loss of function - Can become systemic in severe cases - Chronic progression may mimic low-grade inflammation With decompensation, it can transform into systemic hyperinflammation.	[82]
Systemic Hyperinflammation	A severe, systemic inflammatory response resulting in widespread tissue damage.	- Microcirculatory disorders - Significant systemic damage - Dysfunctional inflammatory response	Results from severe systemic alterations or inflammation dysfunction.	- Refractory shock - Resuscitation syndromes - Acute respiratory distress - Multi-organ failure	[84,85,86,87]
Low-Grade Inflammation (Parainflammation), can be systemic and local. Characteristic variants: meta-inflammation and inflamm-aging.	A chronic, mild inflammation associated with metabolic disorders and aging.	- Chronic exposure to mild damaging factors - Stromal macrophages - Degenerative changes in organs	Responds to conditions like obesity and type 2 diabetes; maintains stable allostasis.	- No barrier function - Generalizes within tissues and body over time - Leads to stable allostasis- Associated with morbid obesity, metabolic syndrome, type 2 diabetes, and other conditions	[88,89,90,91,92,93,94,95,96,97,98,99,100,101,102,103]
Endotheliosis. Key phenomenon Low-Grade Inflammation	A para-inflammatory dysfunction of endothelial cells leading to hypertension.	- Reduced nitric oxide (NO) - Increased vasopressors (e.g., angiotensin II, endothelin I), - impaired barrier function of the endothelial glycocalyx	Contributes to hypertension and atherosclerosis, latent microcirculatory disorders and thrombophilia.	- Spreads through the vascular network - Activates and thins the glycocalyx - Impairs barrier function in arteries - Markers: glycocalyx degradation, activated endotheliocytes, Ang-2, endocan, ET-1, soluble adhesion receptors	[102,103,104,105,106]
Endotheliitis The phenomenon of a focus of canonical inflammation and systemic hyperinflammation	Inflammation of endothelial cells characterized by microthrombosis of postcapillaries and “inflammatory microcirculation”	- Activation of inducible NO synthase - Overproduction of NO - Vasodilation and exudative-vascular response	Promotes vasodilation and localized responses, including inflammatory edema damaged fabrics; can lead to systemic disorders.	- Localized in postcapillary zones - Markers: glycocalyx degradation, - DIC markers, - various endothelial hyperstress products, including soluble adhesion receptors	[102,103,104,105,106,107]
Systemic Low-Grade Inflammation	A chronic systemic inflammation with extensive tissue involvement and dysfunction.	- Reduced capillary density - Microvascular occlusions - Dysfunction in vascular cells	Influences diseases like diabetic kidney disease, osteoarthritis, neurodegeneration.	- Ischemia - Sclerosis and tissue degeneration - Affects organs like kidney, liver, heart, brain, eyes, muscles, joints - Can transform from parainflammation to classical inflammation	[81,108,109,110,111,112,113,114,115,116,117]
Infectious Variants of Low-Grade Inflammation	Variants of low-grade inflammation driven by infectious agents.	- Persistent infection - Chronic immune activation, endotheliosis	Contributes to accelerated aging, sclerotic and degenerative changes in various organs.	- Requires markers of systemic response and endotheliosis - CRP levels (3 to 10 mg/L) insufficient alone - Persistent infection and chronic immune activation	[117,118]

## Data Availability

No new experimental data were created.
